# Case Report: Pneumocystis jiroveci pneumonia: report of five children with the nephrotic syndrome and review of the literature

**DOI:** 10.3389/fped.2025.1687471

**Published:** 2025-12-08

**Authors:** Peitong Han, Xiaoying Yuan, Chunzhen Li, Lei Zhang, Jieyuan Cui

**Affiliations:** Department of Nephrology and Immunology, Hebei Provincial Children’s Hospital, Shijiazhuang, China

**Keywords:** pneumocystis carinii pneumonia, nephrotic syndrome, children, clinical data, five cases

## Abstract

**Objective:**

To retrospectively analyze the clinical characteristics, treatment, and prognosis of five pediatric patients with nephrotic syndrome who developed Pneumocystis carinii pneumonia (PJP) after long-term use of steroids and tacrolimus.

**Methods:**

A review was conducted on five cases of nephrotic syndrome in children who developed Pneumocystis pneumonia after long-term treatment with steroids and tacrolimus. The initial symptoms, early clinical characteristics, and imaging changes were summarized. Among these cases, two were diagnosed through alveolar lavage fluid metagenomic testing and three through sputum metagenomic testing (BGI Genomics Co.), all indicating (pneumocystis carinii pneumonia. All five patients received early treatment with sulfa drugs, with three of them also receiving caspofungin.

**Results:**

One child died and four were discharged with symptomatic improvement.

**Conclusion:**

PJP is a severe opportunistic infection that can progress rapidly and lead to life-threatening respiratory failure, particularly in immunocompromised individuals. Among children with nephrotic syndrome (NS), prolonged exposure to glucocorticoids, tacrolimus, or other immunosuppressive agents markedly increases susceptibility to PJP. Therefore, heightened clinical vigilance, early etiological diagnosis, and prompt initiation of appropriate therapy are essential to improving clinical outcomes in this population.

## Introduction

NS is the most common glomerular disorder in children and is clinically characterized by edema, heavy proteinuria, hypoalbuminemia, and hypercholesterolemia. Glucocorticoids (GCs) remain the first-line treatment for pediatric NS. Approximately 78%–91% of affected children achieve complete remission of proteinuria within 4–6 weeks of GC therapy, a condition defined as steroid-sensitive NS (SSNS). However, 70%–80% of these patients experience at least one relapse, and 25% to 50% develop frequently relapsing (FRNS) or steroid-dependent NS (SDNS) (1). A subset of patients progress to steroid-resistant NS (SRNS), posing a major therapeutic challenge. Children with NS are predisposed to infections due to multiple factors, including reduced serum IgG levels, impaired antibody synthesis, decreased complement components (factors B and D), and the immunosuppressive effects of steroids and other agents. Among these, respiratory tract infections are the most frequent and clinically significant. PJP is a potentially fatal pulmonary infection that primarily affects individuals with compromised immune function. With the increasing use of steroids, immunosuppressants, and biologic therapies for renal and rheumatic diseases, the incidence of PJP in non-HIV immunocompromised patients has been rising (2). The clinical manifestations of PJP are often non-specific in the early stages, which frequently leads to delayed or missed diagnosis. Once established, the disease can progress rapidly, with reported mortality rates ranging from 30% to 50%. Despite its clinical importance, reports of PJP in children with NS remain scarce (3) NS without renal pathology report. The present study retrospectively analyzes the clinical characteristics of five children with NS complicated by PJP who were treated at our center. We summarize their clinical presentations, treatment regimens, diagnostic and therapeutic processes, and outcomes, and provide a brief review of the relevant literature. Our findings aim to enhance clinical awareness and support the early diagnosis and effective management of PJP in pediatric patients with NS.

## Study subjects and methods

We retrospectively analyzed five pediatric patients with nephrotic syndrome (NS) complicated by Pneumocystis carinii pneumonia (PJP) treated at the Department of Renal Immunology, Hebei Children's Hospital, between June 2019 and April 2024. All patients were male and were undergoing treatment with steroids and tacrolimus at the time of onset. This study did involve human subjects and was approved by the ethics committee.

## General information

### Case 1

A 3-year-old boy with nephrotic syndrome (SSNS, FRNS) and no renal pathology received sequential treatment with cyclophosphamide and tacrolimus. In the tenth month of therapy, he developed fever, cough, sore throat, and dyspnea. Laboratory tests revealed leukocytosis with neutrophilia, and chest CT showed diffuse bilateral infiltrates, interstitial changes, pleural thickening, and effusion. Metagenomic analysis of bronchoalveolar lavage fluid confirmed Pneumocystis jirovecii infection, with a tacrolimus trough level of 6.24 ng/mL. Treatment included trimethoprim–sulfamethoxazole (TMP-SMX) and caspofungin, along with broad-spectrum anti-infective therapy, immunoglobulin support, and ventilator assistance. Despite aggressive management, the patient developed persistent high fever and severe hypoxemia, complicated by bilateral pneumothorax, and died despite resuscitation efforts.

### Case 2

A 2-year-old boy with nephrotic syndrome (SSNS, FRNS) and no renal pathology had been receiving steroids and tacrolimus for 11 months when he developed tachypnea. Chest CT showed no significant abnormalities, but bronchoalveolar lavage fluid metagenomic sequencing detected Pneumocystis jirovecii with low read counts, and the tacrolimus trough level was 5.76 ng/mL. Prophylactic TMP-SMX was initiated. One month later, tachypnea recurred, and chest CT revealed multiple linear opacities and interstitial changes in both lungs. A diagnosis of PJP with diffuse interstitial lung disease was made. Therapeutic-dose TMP-SMX was administered for one week, leading to clinical and radiologic improvement. The patient continued prophylactic TMP-SMX for over four months without relapse.

### Case 3

A 4-year-old boy with secondary steroid-resistant nephrotic syndrome (SRNS) was diagnosed with minimal change disease on kidney biopsy and treated with tacrolimus. In the 22nd month of therapy, he developed tachypnea, cough, and wheezing. After three days of empirical anti-infective therapy, fever and cough worsened. Metagenomic testing of sputum confirmed Pneumocystis jirovecii infection. Chest CT revealed diffuse bilateral infiltrates, interstitial changes, and pleural effusion. The tacrolimus trough level was 7.56 ng/mL. Therapeutic-dose TMP-SMX was given for five days, but radiologic findings deteriorated; caspofungin was then added for one week, resulting in marked improvement. Maintenance-dose TMP-SMX was continued for four months without recurrence.

### Case 4

A 6-year-old boy with nephrotic syndrome (SSNS, SDNS) received steroids and tacrolimus, and a single dose of rituximab during treatment. In the 37th month of therapy, he developed high fever and cough. Despite empirical anti-infective treatment, fever persisted, and on the fourth day he developed dyspnea and oxygen desaturation. Metagenomic analysis of sputum identified Pneumocystis jirovecii. Chest CT revealed diffuse high-density opacities, bilateral pleural effusion, and exudative changes in the chest wall. The tacrolimus trough level was 4.97 ng/mL. After four days of therapeutic-dose TMP-SMX without improvement, tacrolimus was discontinued, and caspofungin was added for one week, leading to clinical resolution. Maintenance-dose TMP-SMX was continued for four months, then tapered off, with no recurrence.

### Case 5

A 3-year-old boy with nephrotic syndrome (SSNS, FRNS) received steroids combined with tacrolimus. In the 12th month of therapy, he developed tachypnea, and on the fifth day developed fever and cough, without significant respiratory distress or hypoxemia. Metagenomic analysis of sputum confirmed Pneumocystis jirovecii infection. Chest CT revealed multiple linear opacities and diffuse interstitial lung lesions. The tacrolimus trough level was 5.88 ng/mL. After two weeks of therapeutic-dose TMP-SMX, repeat chest CT showed improvement, and the patient was discharged. However, failure to continue prophylactic TMP-SMX led to recurrence of tachypnea one month later. Repeat CT again revealed diffuse interstitial lesions. After one week of retreatment with TMP-SMX, symptoms improved markedly, and follow-up CT showed near-complete resolution. Maintenance-dose TMP-SMX was continued for three months, then gradually discontinued, with no relapse.

## Summary

All five cases involved primary simple nephrotic syndrome: three were frequently relapsing NS, one was steroid-resistant NS with biopsy-proven minimal change disease, and one was steroid-dependent NS. All patients had received steroid therapy and were on tacrolimus at the onset of PJP; one had also received rituximab (Table 1).

**Table 1:** Basic data of 5 children with NS complicated with PJP.

Item	Case 1	Case 2	Case 3	Case 4	Case 5
Fever	High fever with chills	No	High fever with chills	High fever	High fever
Cough	Mild	Mild	Mild	Mild	Mild
Onset of dyspnea	Yes	No	Yes	Yes	Yes
Decreased blood oxygen saturation	Yes	No	No	Yes	Yes
Rales in lung	Yes	No	Yes	Yes	Yes
First symptoms	Onset of dyspnea	Shortness of breath	Fever	Cough	Cough
Dureation of fever(days)	11	0	9	10	3

### Clinical manifestations

The initial symptoms of the five patients were as follows: two presented with fever and cough, one with fever and rapid breathing, and two with rapid breathing. As the disease progressed, all patients exhibited varying degrees of fever, cough, and dyspnea. The highest recorded body temperature ranged from 39.0 to 40.2°C. The cough was predominantly dry and accompanied by varying degrees of fatigue and shortness of breath. Difficulty breathing occurred one to seven days after the onset of fever, and two patients had blood oxygen saturation levels below 90%. Clinical signs varied among patients: one had tachypnea, three showed intercostal retractions, and three had moist rales on auscultation (Table 2).

**Table 2:** Clinical characteristics of PJP in 5 children with NS.

Chest CT	Bilateral lung parenchyma, interstitial and pleural lesions	Bilateral lung parenchyma and interstitial lesions	Bilateral lung parenchyma, interstitial and pleural lesions	Bilateral lung parenchyma, interstitial and pleural lesions	Bilateral interstitial lesions
Pathogen detection	Metagene detection of bronchoalveolar lavage fluid	Metagene detection of bronchoalveolar lavage fluid	Metagene detection of sputum	Metagene detection of sputum	Metagene detection of sputum
Tac trough concentration (ng/mL)	6.24	5.76	7.56	4.97	5.88
Treatment	TMP-SMX, Caspofungin	TMP-SMX	TMP-SMX, Caspofungin	TMP-SMX, Caspofungin	TMP-SMX,
Treatment results	Dead	Cure	Cure	Cure	Cure

### Auxiliary examinations

Laboratory Indicators: All patients showed significantly elevated levels of C-reactive protein and procalcitonin. Three patients had markedly elevated white blood cell counts, predominantly neutrophils.

**Figure 1:**
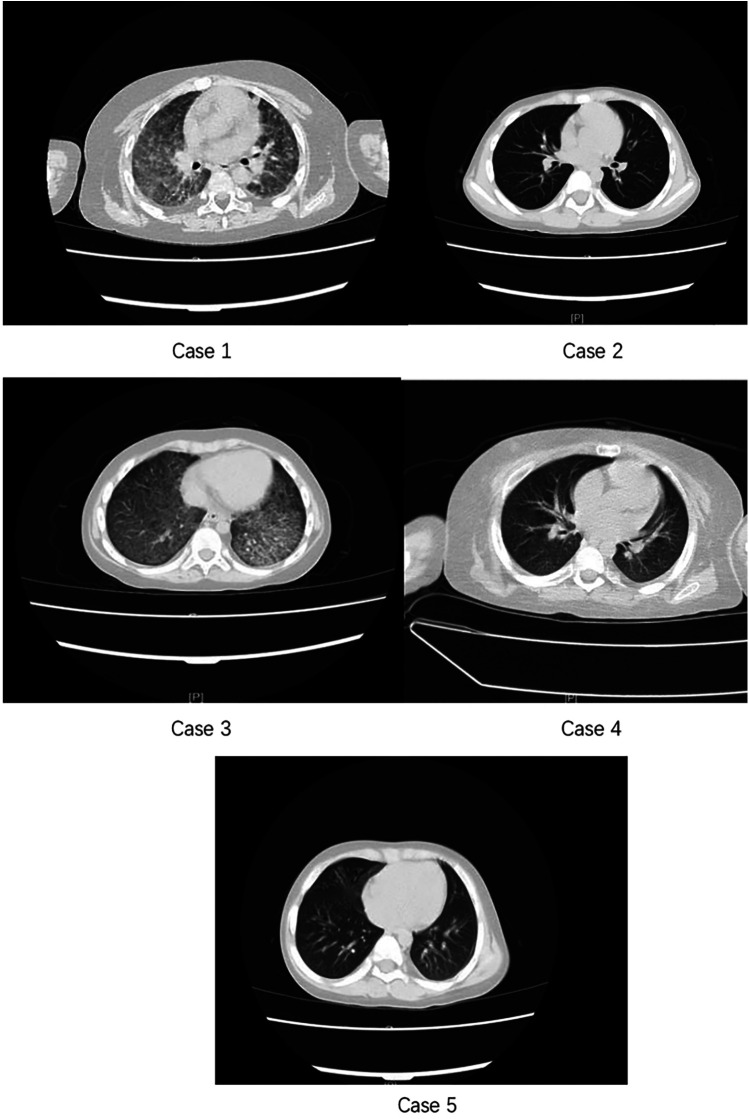
Chest computed tomography scans of five children with nephrotic syndrome complicated by Pneumocystis jiroveci pneumonia (Cases 1–5). The images show bilateral interstitial and alveolar infiltrates of varying extent, with some cases demonstrating pleural effusion and more extensive parenchymal involvement.

Imaging Findings: Early chest CT scans were performed for all five patients, revealing diffuse bilateral pulmonary lesions. Four cases demonstrated bilateral interstitial involvement, with one case primarily exhibiting interstitial lung disease. Four patients presented with pleural effusion and varying degrees of pleural changes, including three with pleural thickening and one with pleural exudation (Figure 1, see Cases 1–5).

Pathogen Examination: All five patients underwent pathogen testing. Metagenomic analysis of bronchoalveolar lavage fluid was performed in two cases, and metagenomic analysis of sputum samples in three cases; Pneumocystis jirovecii was identified in all patients.

### Treatment process

All five patients were undergoing treatment with steroids and tacrolimus at the onset of PJP. The original medication dosages were maintained in all cases, except one where tacrolimus was discontinued due to worsening lung lesions. Upon diagnosis, all patients were immediately treated with trimethoprim-sulfamethoxazole (TMP-SMX) (TMP 15–20 mg•kg^−1^•d^−1^, SMX at a dosage of 75–100 mg·kg⁻¹·day⁻¹, administered orally in three divided doses for a total duration of 2–3 weeks. Three patients with more severe lung lesions received additional caspofungin treatment (initial dose 70 mg/m^2^ intravenously, followed by 50 mg/m^2^ daily).

### Treatment outcomes

Of the five patients, one died due to severe lung lesions, while the remaining four were clinically cured and did not experience recurrence during a follow-up period of over six months, all patients had normal renal function and no recurrence of NS disease.

## Discussion

Nephrotic syndrome (NS) is one of the most common kidney diseases in pediatrics, characterized by abnormalities in the glomerular filtration membrane, leading to edema, heavy proteinuria, hypoalbuminemia, and hypercholesterolemia. The etiology includes genetic, primary, and secondary causes, with primary NS being the most common.

Pneumocystis carinii pneumonia (PJP) is an opportunistic infection that predominantly affects individuals with congenital/acquired immune deficiencies or those undergoing long-term immunosuppressive therapy. Its early symptoms are insidious, with rapid progression, making it prone to misdiagnosis or missed diagnosis due to the lack of typical clinical manifestations. Some researchers suggest renaming the species infecting humans from Pneumocystis carinii (PC) to Pneumocystis jirovecii (PJ) to distinguish it from the species infecting rats (4).

Glucocorticoids (GCs) are the first-line treatment for pediatric NS. Patients with FRNS and SDNS often require additional immunosuppressants (such as tacrolimus, cyclophosphamide, cyclosporine A) and biologics (such as rituximab) to maintain remission (5). Children with NS exhibit significant immune dysfunction, with serum IgG levels significantly lower and IgM levels higher than those in healthy children. Children on long term high dose immunosuppression can further exacerbate immune deficiencies, increasing the risk of infections, including opportunistic infections (6).

Tacrolimus is a calcineurin enzyme inhibitor which, in addition to its immunosuppressive effects, has the advantages of stabilising the podocyte cytoskeleton, promoting podocyte repair and improving the symptoms of proteinuria. However, it also inhibits T-cell activation and proliferation, reduces B-cell antibody production, and significantly suppresses cellular and humoral immunity. The recommended blood concentration is 3–7 ng/mL. Tacrolimus, a calcineurin inhibitor widely used in pediatric nephrotic syndrome, may predispose patients to Pneumocystis jirovecii pneumonia (PJP) even at therapeutic trough levels. This risk is linked to tacrolimus-induced suppression of T-cell activation and cytokine production, key components of host defense. Concurrent corticosteroid therapy may further enhance immunosuppression. In this series, PJP occurred despite therapeutic tacrolimus levels, indicating that adequate drug monitoring does not fully prevent opportunistic infections. Additionally, rituximab, a B-cell–depleting monoclonal antibody, may increase infection risk through hypogammaglobulinemia and delayed immune recovery. These observations underscore the need for careful monitoring and consideration of prophylactic measures during combined immunosuppressive therapy. This study reviewed five pediatric NS patients on long term high dose immunosuppression, all of whom presented with PJP, for periods ranging from 10 to 37 months, and all of whom had tacrolimus blood levels within the normal range. One patient had been treated with a single dose of rituximab, and one patient had serum IgG levels below normal. Rituximab treatment for NS has been reported to be complicated by PJP, suggesting that patients receiving combination therapy with biologics should be closely monitored for potential opportunistic infections (6, 7).

Patients with PJP-complicated NS face serious complications with rapid progression that can be life-threatening in the short term. Immediately after the onset of symptoms (8), five patients were treated with TMP-SMX based on signs of lung infection and CT findings. Three patients received additional caspofungin therapy due to the severity of their condition, but one patient died due to severe lung lesions. Severe PJP infections have been reported in NS patients treated with biologics, and those with additional infections appear to have a higher mortality rate (6). Some studies have suggested that nasal transmission between transplant patients is the primary route of transmission; however, the life trajectories of our five patients did not overlap during their illnesses (9).

Pathogen testing is the gold standard for the diagnosis of PJP, and early detection is essential, especially metagenomic analysis of sputum and bronchoalveolar lavage fluid helps in early diagnosis. Sputum metagenomic analysis is not entirely sensitive; three of our five patients had positive sputum Metagenomic analyses, and two required bronchoalveolar lavage metagenomic analysis to confirm the diagnosis of Pneumocystis carinii infection. Bronchoscopic biopsy improved the overall detection rate for diagnoses other than PJP. Bronchoalveolar lavage should be performed in patients in whom the diagnosis cannot be confirmed by sputum analysis (10). Open lung biopsy is rarely required because of the effectiveness of sputum and bronchoscopy, and its detection rate may be related to the primary disease, disease progression, and testing technique.

In children on long-term steroid and tacrolimus therapy, active control of the disease, close monitoring of disease changes, regular clinical assessments and imaging studies as necessary, in addition to antifungal therapy, are required. TMP-SMX is effective in preventing PJP (11), therefore, it is worth considering the prophylactic use of sulfonamides in this patient population. Prophylactic administration of trimethoprim–sulfamethoxazole (TMP-SMX) is highly effective in preventing Pneumocystis jirovecii pneumonia (PJP) in immunocompromised pediatric patients, including those receiving corticosteroids and calcineurin inhibitors such as tacrolimus. Moreover, remission of nephrotic membranous glomerulonephritis after high-dose trimethoprim-sulfamethoxazole treatment for Pneumocystis jiroveci pneumonia has been reported (12). The benefits include a significant reduction in PJP incidence and associated morbidity and mortality. However, TMP-SMX carries potential risks, including hypersensitivity reactions, hematologic toxicity, and renal impairment, which require monitoring during therapy. On balance, the protective effect against a life-threatening opportunistic infection generally outweighs these risks. Current evidence supports initiating prophylactic TMP-SMX in high-risk children, particularly those receiving combined immunosuppressive regimens, with dose adjustment based on weight and renal function (4). Close monitoring for adverse effects is essential, and therapy should continue throughout the period of immunosuppression to maximize protection. However, the prophylactic use of TMP-SMX carries certain risks, such as rash, gastrointestinal symptoms, and the induction of drug-resistant bacteria. Therefore, when deciding whether to recommend the prophylactic use of sulfonamides, the patient's physical condition should be assessed and the corresponding side effects should be informed in advance.

Because this study reported only five cases and lacked a control group of children with PJP but without NS, the generalizability of our findings is limited. We will continue to collect more samples and seek multicenter collaboration.

## Data Availability

The original contributions presented in the study are included in the article/Supplementary Material, further inquiries can be directed to the corresponding author.
